# Accelerated para-articular osteochondroma formation within the knee: a case report

**DOI:** 10.1186/1757-1626-1-6

**Published:** 2008-05-12

**Authors:** Michael R Carmont, Sian Davies, Daniel Gey van Pittius, Robin Rees

**Affiliations:** 1 The Departments of Trauma and Orthopaedic Surgery, The University Hospital of North Staffordshire, Stoke-on-Trent, UK; 2 Histopathology, The University Hospital of North Staffordshire, Stoke-on-Trent, UK

## Abstract

**Introduction:**

Intra-articular osteochondroma and acoustic neuroma are rare entities.

**Case presentation:**

We report the rare occurrence of a para-articular osteochondroma of the knee developing over short duration, 5 months, following minor injury.

**Conclusion:**

Predisposition to heterotrophic ossification after previous neurosurgery and a second acoustic neuroma may have accelerated the growth of this benign tumour. The development of these two rare entities suggests they may be associated.

## Introduction

Anterior knee pain is a common symptom and may be caused by pathology found in the patellofemoral joint or the infrapatella fat pad. Intra-articular osteochondroma and acoustic neuroma are rare entities. We report the development of an intra-articular osteochondroma which may be associated with the growth of an acoustic neuroma.

Predisposition to heterotrophic ossification after previous neurosurgery or the recent development of a second acoustic neuroma may have accelerated the growth of this benign tumour.

## Case Report

A sixty-one year old gentleman complained of two-day history of increased pain and swelling of his right knee. Five months earlier he had sustained a twisting injury to his knee while playing golf. Following the injury he was able to complete his game and there was minimal swelling. He was aware of a continued dull ache to the front of his knee which was worse on going up and down stairs.

He previously had a nephrectomy for renal cell carcinoma. He also had an acoustic neuroma excised 20 years previously. Eleven-months prior to the knee discomfort he was noted to have developed another acoustic neuroma in the opposite side. There was no family history of neuromata or atypical metaplasia.

The knee had lost 10 degrees of extension and flexed to 100 degrees. The opposite knee flexed to 120 degrees. The tissues around the patellar tendon were swollen and there was an effusion. The medial joint line and medial femoral condyle were tender. The collateral and cruciate ligaments were stable.

**Figure 1:**
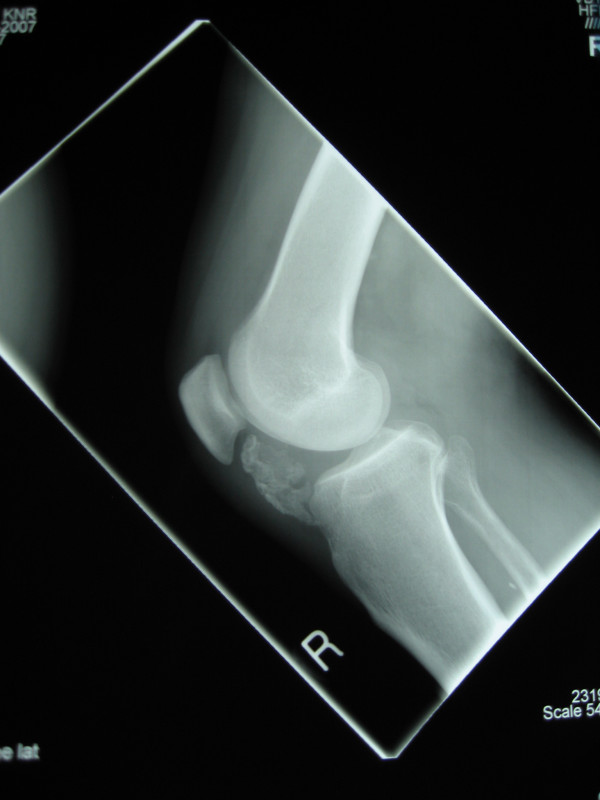
Lateral radiograph of the knee demonstrating ossification in the peritendinous tissues.

**Figure 2:**
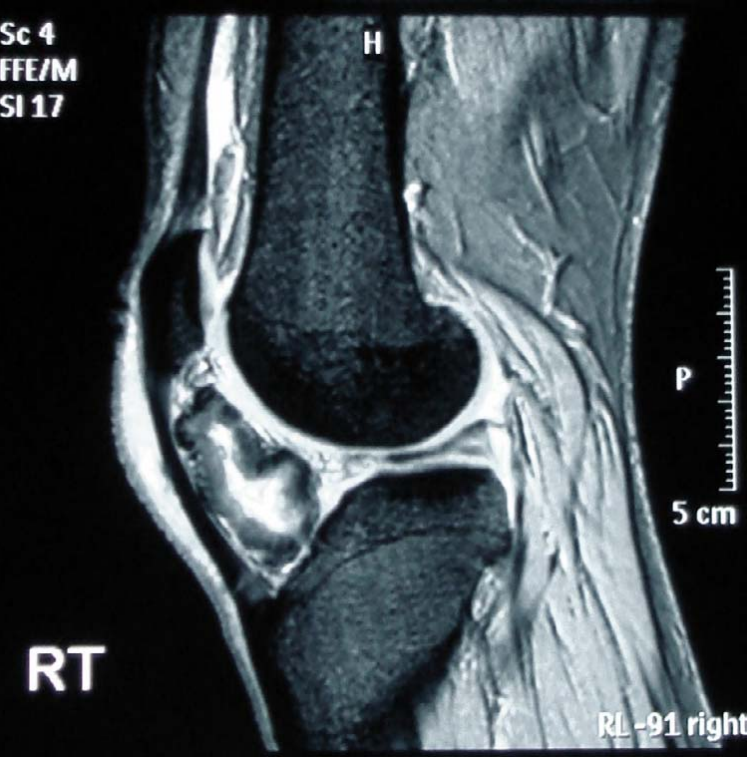
**Sagittal magnetic resonance scans of the knee.** The ossified tissues have low signal and appear dark on both T1 and T2 weighted images. The increased bright signal on T2 shows the oedema within the fat pad. The fat pad has high signal on the T1 image.

Plane radiographs demonstrate peritendinous calcification deep to the patellar tendon, with no degenerative features in either the tibio-femoral or the patello-femoral joints (Figure 1). Magnetic resonance imaging shows the ossified tissues as low signal and appears dark on both T1 and T2 weighted images. The increased bright signal on T2 shows the cartilage within the osteochondroma and oedema within the fat pad. The fat pad has high signal on the T1 image (Figure 2). The lesion was located in the extra-synovial tissues of the fat pad.

**Figure 3:**
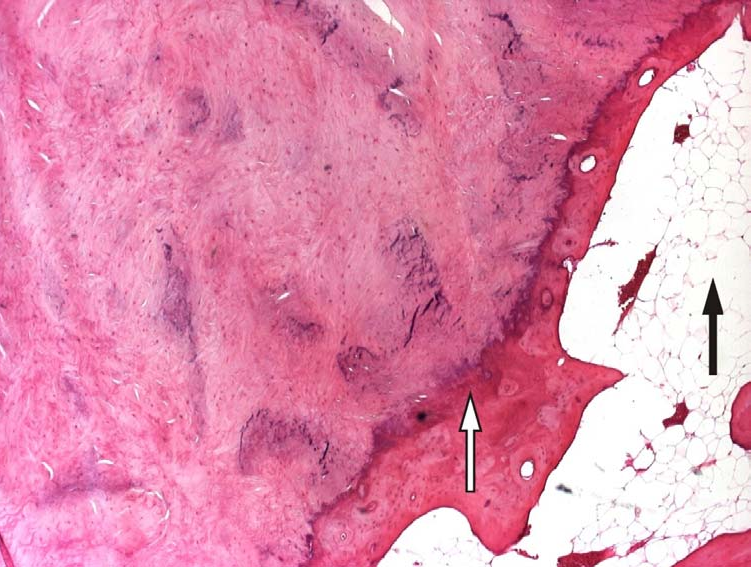
Histopathology slide (magnification × 40) showing the enclosing fat cells of the infra-patellar fat pad (solid arrow), a peripheral rim of woven immature bone (hollow arrow) enclosing cartilage cells.

Arthroscopy confirmed normal menisci and no evidence of chondral injury but there was thickening to the infra patellar fat pad. The calcified lesion within the fat pad was excised through a lateral para-patellar approach using a midline incision. The lesion was 45 mm in diameter, and had been completely excised. On microscopic examination it was composed of acellular dense hyalinised fibrocollagenous tissue with evidence of focal ossification, cartilaginous metaplasia and calcification. Figure 3, magnification × 40, with haematoxylin and eosin staining, shows the enclosing large clear fat cells of the infra-patellar fat pad (solid arrow). There is a peripheral rim of woven immature bone, seen as haphazard pink cells (hollow arrow) enclosing the fibrocollagenous centre.

## Discussion

Intra-articular tumours occur rarely and when they do occur can be found within the joint or extra-synovial tissues. Solitary benign lesions were termed para-articular chondroma or intracapsular chondromas by Jaffe in 1958. The knee is most commonly affected although this entity has been described in other joints [1,2]. The morphological features of these entities are better described as para-articular osteochondromas. Although the WHO definition of an osteochondroma is a cartilage capped bony projection arising from the external surface of the bone containing a marrow cavity that is continuous with that of the underlying bone. These have been further categorised into those found within the infrapatella fat pad and those as pedunculated lesions within the knee joint itself.

Thickening of the infrapatella fat pad following minor injury has been described by Hoffa. Calcification and ossification of the fat pad may be the end stage of this process [3]. The association between minor injury and the formation of a para-articular osteochondroma has also been described [4]. The rapid development of bone within soft tissues is termed heterotrophic ossification (HO). It is well appreciated that HO can occur following central nervous system injury including iatrogenic insult, although its aetiology is unknown. A search of the literature has not revealed an upper duration of this association although we appreciate that it is likely to be short term. The knee injury with development of the osteochondroma has occurred only several months after the diagnosis of the second acoustic neuroma. We appreciate that it is possible that the patient may have had the lesion for many years and however he was aware of it several months after his injury. Our patient clearly recalled suffering a minor knee injury and subsequently noted anterior knee discomfort and then gradually developed fullness to the front of his knee over five months. We are unaware of any association between osteochondroma and acoustic neuroma but since both entities are rare this is unlikely to be a coincidence.

There are 31 cases reported in the literature, described in 2 reviews [1,2] and an additional 6 individual case reports [3,5-10]. In the majority of cases the lesions were slow growing, with patients reporting symptoms for many years, although 3 patients reported symptoms from 2 months to a year. There were no cases reported in skeletally immature patients and most were in or beyond the fifth decade.

Since para-articular osteochondroma formation is rare it is impossible to say whether prompt debridement of the infra-patella synovium and fat pad may have prevented or delayed its formation. In any tumour, there is potential for recurrence and malignant transformation however we appreciate that in this case the risk is exceptionally small. The patient has been advised to seek further medical attention in there is recurrence of the lesion.

## Conclusion

We report the rare occurrence of a para-articular osteochondroma of the knee developing over short duration, 5 months, following minor injury. Predisposition to heterotrophic ossification after previous neurosurgery and a second acoustic neuroma may have accelerated the growth of this benign tumour. The development of these two rare entities suggests they may be associated.

## Abbreviations

HO: Heterotrophic ossification; WHO: World Health Organisation.

## Competing interests

The authors declare that they have no competing interests.

## Authors' contributions

MRC wrote the case report, performed the literature review and obtained written consent. SD performed the literature search and assisted with writing the paper. DGvP obtained the histopathological photographs for the study. RR conceived the study and helped to draft the manuscript. All authors have read and approved the final manuscript.

## Consent

Written informed consent was obtained from the publication of this case report and accompanying images. A copy of the written consent is available for review by the Editor-in-Chief of this journal.
